# Incidence, diagnostics and treatment algorithm of nerve lesions after traumatic shoulder dislocations: a retrospective multicenter study

**DOI:** 10.1007/s00402-020-03348-z

**Published:** 2020-01-24

**Authors:** T. M. Tiefenboeck, J. Zeilinger, M. Komjati, C. Fialka, S. Boesmueller

**Affiliations:** 1 Division of Trauma Surgery, Department of Orthopaedics and Trauma Surgery, Medical University of Vienna, Waehringerguertel 18-20, Vienna, 1090 Austria; 2 AUVA Trauma Center Vienna Meidling, Kundratstraße 37, Vienna, 1120 Austria; 3 Faculty of Medicine, Sigmund Freud University, Freudplatz 1, Vienna, 1020 Austria

**Keywords:** Shoulder dislocation, Plexus brachialis lesion, Nerve lesion

## Abstract

**Background:**

The shoulder is the joint most prone to dislocating in the human body and accounts for 45% of all dislocations. In addition to ruptures of the soft tissue and bony injuries, lesions to vascular structures as well as the brachial plexus and its corresponding nerves might occur. With an incidence of up to 65%, nerve lesions are frequently reported after shoulder dislocations. The aim of this study is to obtain information on epidemiology, diagnostics, treatment and duration until remission or late sequelae after shoulder dislocation and concomitant nerve injury in a large patient cohort.

**Methods:**

The patient cohort consisted of 15,739 patients from three centres who had sustained a shoulder dislocation. All patient files were searched for concomitant injury of the brachial plexus or its corresponding nerves. For epidemiological data analysis, demographic data, clinical follow-ups, electromyography and nerve conduction velocity were evaluated.

**Results:**

In total, 60 patients (32 males, 28 females) with a mean age of 60 years (range 19–88 years) met the inclusion criteria. In the majority of patients (*n* = 51), the trauma mechanism was a trivial fall on the outstretched arm. The most frequent dislocation direction was anterior-caudal in 61.6%, followed by strictly caudal in 16.6%. The brachial plexus was injured in 46 patients (76.6%) and isolated nerve damage was documented in 14 patients (23.3%). Electroneurographic examinations were performed in less than half of the patients (38.3%).

**Conclusion:**

A combination injury of shoulder dislocation and plexus lesion may occur at any age and sometimes has a poor outcome. Electroneurographic examinations should be implemented when managing these patients as a cost-effective and supportive examination.

**Level of evidence:**

Level IV, retrospective study.

## Introduction

The shoulder is considered to be the joint with the highest mobility in the human body [9]. This allows a wide range of motion, which might compromise stability and, therefore, make it prone to dislocations [22]. With a reported prevalence of 1.7%, it is the most frequently dislocated major joint [21]. With regards to direction of dislocation, the anterior dislocation is the most frequent with a prevalence of up to 95% [8,14]. Young male patients are affected most commonly with an incidence of 40.4 in 100.000 [21].

Usually, the injury pattern is an external rotation and hyperextension of the arm, levering the humeral head out of the glenoid [8]. This can lead to acute and chronic shoulder injuries that need to be treated. Bankart lesions, rotator cuff tears (RCT), impaction fracture of the humeral head (Hill-Sachs lesion), or of the greater tuberosity, are likely accompanied with this type of injury [8,14].

Due to the close anatomical proximity of the shoulder joint and the brachial plexus, nerve injuries are reported with an incidence of 21–65% [2,7,13]. In nearly all cases, traction to the nerve by downward displacement of the humeral head is reported. In most cases, the axillary nerve is damaged due to its fixed position, leading to a decrease of sensation over the inferior deltoid muscle (regimental badge area) of the affected arm.

The diagnosis usually begins with a careful history and clinical examination to determine a shoulder dislocation. X-rays in three planes are indispensable to obtain more precise information about the direction of dislocation and any associated bony injuries. When suspecting concomitant injuries, arthro-MRI, MRI and CT are used. If clinical signs for suspected sensory deficits or plexus lesions are present, further diagnostics including plexus MRI and/or ultrasound as well as electromyography (EMG) and nerve conduction velocity (NCV) are necessary [19].

Treatment of these lesions mostly consists of conservative therapy in the sense of physical therapy, lymph drainage, ultrasound therapy and treatment with vitamin B preparations to support nerve regeneration. In more complex cases, i.e. complete nerve disruption or root rupture, neurosurgery may be necessary [18,19].

Thus, the aim of this study was to present incidence, diagnostics, treatment and outcome of injury of the brachial plexus or its corresponding nerves after traumatic shoulder dislocation in a large retrospective multicentre trial to offer more coherent information for affected patients.

## Materials and methods

After obtaining approval of the institutional ethics committee (No. 1348/2018), this retrospective multicenter study was performed. The participating centres consisted of the Medical University of Vienna (MUVI), Department of Orthopaedics and Traumatology, as well as the AUVA Trauma Centres Meidling and Lorenz Böhler. All patients who sustained a shoulder dislocation with a concomitant plexus injury or isolated nerve lesion between January 2000 and December 2016 were included. Patients’ data were searched for the ICD 10 code for shoulder dislocation in combination with a plexus lesion or isolated nerve lesion.

Inclusion criteria were as follows: (1) shoulder dislocation and plexus lesion; (2) shoulder dislocation and isolated nerve lesion and (3) documented treatment details to final follow-up. Exclusion criteria were as follows: (1) shoulder dislocation without plexus or nerve lesions; (2) incomplete data set and (3) missing documented treatment details to final follow-up.

The main parameter was the clinical outcome [ROM, patient satisfaction and activities of daily living (ADLs)] at final follow-up (at least 1 month after dislocation) including a neurological examination, NCV and/or EMG investigation. Additionally, sex, age, direction of dislocation, number of dislocations, time until reduction, technique of reduction, concomitant injuries, treatment and length of treatment were evaluated. Patients’ clinical outcome was classified as not impaired (free ROM, subjectively satisfied, no problems in ADLs), mild impairment (ROM up to 90° abduction and flexion possible, subjectively not satisfied, no problems in ADLs) and impairment (limited ROM, subjectively not satisfied, problems in ADLs).

The treatment of incomplete nerve lesions consisted of supportive therapy including physiotherapy, electrotherapy and lymph drainage. In addition, vitamin B was prescribed to support the regeneration of nerve tissue.

**Fig. 1:**
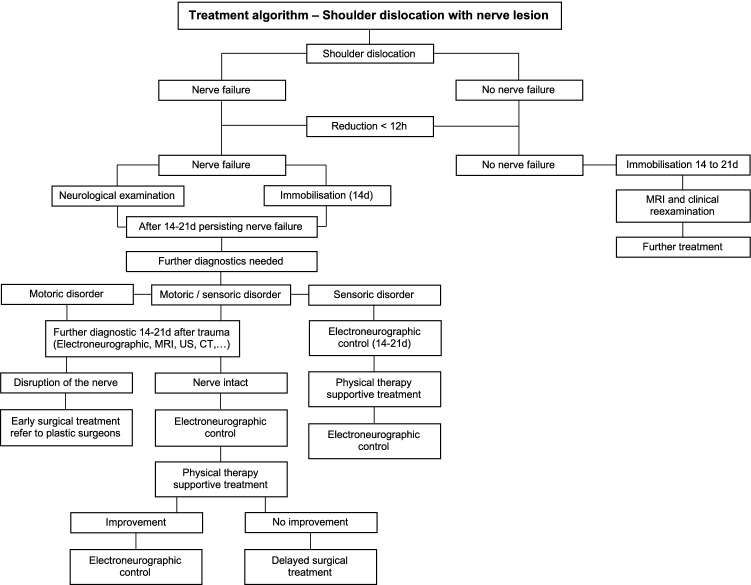
Diagnostic and treatment algorithm based on literature and clinical experience

To document the healing process, EMGs were carried out every 2–3 weeks to monitor the response to therapy. Due to the long time span of this study, the treatment algorithm, especially due to superior diagnostic methods, changed over time. Today, in all of our patients with neurologic disorders especially with persisting nerve disorders, electroneurographic diagnostics as well as plexus MRI and/or ultrasound is routinely carried out. A diagnostic and treatment algorithm based on the literature [6,15,18-20] and our clinical experience is presented in Fig. 1.

In cases of severe nerve lesions, such as root rupture, patients should be referred to neurosurgery or plastic surgery for further treatment [11], which was not necessary in our series.

## Statistical analysis

Descriptive data (mean, median, range, proportions) are reported for the entire patient cohort. Statistical analysis focused on clinical outcome over a set time period. Therapeutic variables (type of reduction, number of dislocations, direction of dislocation, time until reduction, primary or secondary nerve lesion, treatment, additional injuries, treatment and length of treatment) and demographic variables (sex, age and follow-up) were examined. The Chi^2^ test was used to test significances between categorical variables. Cramér’s *V* was used to measure associations between two nominal variables, giving a value between 0 and + 1 (inclusive). It is based on Pearson’s Chi-squared statistics.

All calculations were made using Microsoft Excel^®^ and SPSS^®^ software (Version 25.0, SPSS Inc., Chicago, IL, USA).

## Results

### Patients

A total of 15,739 patients with shoulder dislocations were available for study inclusion. After exclusion, 60 patients (16 out of 5840 MUVI; 10 out of 3223 AUVA Trauma Center Vienna Lorenz Böhler and 34 out of 6676 AUVA Trauma Center Vienna Meidling) with shoulder dislocations and associated plexus lesions or isolated nerve lesion were included in this study, representing an overall incidence of 0.4%.

There were 32 men (53.3%) and 28 women (46.7%) with a mean age of 60 years (range 19–88 years; STD 18 years). Seventy percent of patients were aged over 70 years and were female. Overall, plexus- and nerve lesions occurred more frequently in older patients, with nearly 80% being over the age of 52 years at the time of injury. In total, two patients (3%) presented with signs of hyperlaxity at time of dislocation. In another four people, hyperlaxity was observed during the follow-up period.

### Injury pattern

A fall on the outstretched arm was the most frequent injury pattern, occurring in 85% (*n* = 51). Falls while horseback riding (*n* = 1), skiing (*n* = 4) or from the ladder during housework (*n* = 3) were also documented. Other causes were increased pressure on the shoulder joint while playing football (*n* = 3) or moving a heavy armchair (*n* = 1). Increased tension was another trauma mechanism described, for example from a handbag snatch or pulling on a heavy object (*n* = 2).

### Direction of dislocation

The humeral head most frequently dislocated in the antero-caudal direction (*n* = 40; 67%), followed by caudal dislocation, which was documented in 10 patients (17%) and lastly the posterior dislocation, which occurred in two patients (3%). The direction of dislocation had no impact on the occurrence of a nerve/plexus lesion (Cramers *V* = 0.051). The direction of dislocation did not influence the clinical outcome (*p* = 0.324).

In eight patients (13%), the direction of dislocation could not be determined due to missing X-ray or CT images.

In 58 patients (96%), the first dislocation led to a plexus injury. Six patients (10%) presented with recurrent shoulder dislocations or a subluxation phenomenon during the follow-up period. Two of the affected patients (3.3%) had already sustained a previous shoulder dislocation and in four patients (6.6%), a recurrent shoulder dislocation was documented. Re-dislocations occurred after an average of 4 months after the first dislocation.

### Nerve lesions

A total of 46 patients (76.6%) sustained a brachial plexus injury and 14 patients (23.3%) presented with peripheral nerve injuries. The axillary nerve was injured in isolation in eight cases (13.3%). The radial, ulnar and median nerves were each affected twice (3.3%).

A motor deficit was present in 35 patients (58.3%) and a sensory deficit in 52 patients (86.6%) at the time of admission. Thirty-one patients (51.6%) suffered from a combination of both. Four of these patients (6.6%) had only motor and 21 only sensory deficits (35%).

An electrographic examination consisting of either EMG or NCV was performed in 23 patients (38.3%) during the course of the treatment process. EMG was performed in 20 patients and NCV in 23 patients.

### Therapy

In all patients, initial reduction was performed within 12 h after injury in one of the participating hospitals. All patients underwent subsequent immobilization for 2–3 weeks depending on patient age, as well as physiotherapy. In 5 out of 60 patients (8%), surgery was necessary because of additional pathologies, in two patients, vascular surgery due to axillary artery lesions had to be performed immediately. In three patients, shoulder arthroscopy was necessary because of recurrent instability. None of the patients had to be treated operatively for nerve pathologies. Neurobion^®^ (vitamin B1 and B6) was administered to all patients as supportive therapy; however, evidence of this supportive treatment is lacking in humans. At present, a few studies describing the potential of vitamin B in nerve recovery in animals exist; however, this effect has yet to be proven in human studies [16,23,24].

### Concomitant pathologies

Abruption of the greater tubercle was the leading concomitant injury in this study, with 23 cases (38.3%). Lesions of the rotator cuff occurred in seven patients (11.6%). In three patients (5%), a Bankart lesion was detected; in one patient (1.6%), an abruption of the coracoid process and in two patients (3.3%), combination injuries were reported. A terrible triad (anterior shoulder dislocation, rotator cuff tear and brachial plexus lesion) was present in six cases (10%).

### Outcome

At final follow-up (mean 7 months), 25 patients (41.6%) suffered from motor deficits and 21 from sensory deficits (35%). Ten of these (16.6%) presented with combined deficits. An isolated sensory disorder was present in 11 patients (18.3%) at final follow-up. An isolated motor function disorder was present in 15 patients (25%). The final follow-up examination was performed after a mean of 7 months (range 1–86 months, STD 13 months).

At final follow-up, 26 patients (43%) presented with severe impairment and 15 patients (25%) with mild impairment regarding ADLs. A full recovery with regard to clinical outcome was recorded in 19 patients (32%) at final follow-up.

## Discussion

Summarizing our results, we present an overall incidence of 0.4% of injuries to the brachial plexus or its corresponding nerves after shoulder dislocation with reference to the three largest trauma hospitals in Vienna. 50% of the brachial plexus lesions and 57% of the single nerve lesion deficits were present at latest follow-up, showing a fairly high rate at the end of follow-up. In the current literature, there exist only few studies, including case reports or case series, dealing with lesions of the brachial plexus and its corresponding nerves after shoulder dislocations [8].

With 60 patients, our study represents the largest patient cohort investigating treatment and outcome of this rare injury combination [1,7,8,13,17,25,26]. Overall, 46 patients (76.6%) suffered injury to the brachial plexus and 14 patients (23.3%) presented with peripheral nerve lesions. The axillary nerve was most commonly affected, which is in line with the current literature [5].

Peripheral nerve injuries have a potentially devastating impact on quality of life, resulting in severe disability with substantial social and personal cost [12].

In 36 of the 60 patients (60%), the initial motor or sensory deficit was still present at final follow-up, demonstrating the difficulty of treating these lesions successfully. In the course of this study, isolated sensory disorders were detected in 11 patients (18.3%) at the end of therapy. An isolated motor function deficit (without sensory deficits) was found in 15 patients (25%). In 10 patients (16.6%), both sensory and motor deficits were documented at completion of therapy. Brachial plexus injuries needed in mean 7 months of therapy compared to 5 months when a single nerve was affected. However, in 50% of the brachial plexus lesions and in 57% of the single nerve lesions, neurological deficits were present at latest follow-up.

In the current literature, the prognosis of spontaneous recovery after brachial plexus injury following anterior shoulder dislocation is documented to be favourable [8]. However, motor and sensory deficits are not separated. A distinction is mainly only made between restoration of the norm or further existing limitation. Summarizing, the literature describes a good remission rate of symptoms after shoulder dislocation [8,27], which stands in contrast to our data where persistent nerve disorders were observed in 35%. Therefore, it needs to be discussed whether this discrepancy is caused by the low number of included patients in the discussed studies, causing the remission rate to be clearly overestimated. Another explanation may be that our patients collective were overall older compared to other collectives in the literature [3,28]. However, it is known that older people are prone to suffer plexus or nerve injuries more frequently after shoulder dislocations, caused mainly by concomitant injuries such as fractures or haematomas [4,10,27]. In a study by Kosiyatrakul et al. [8], a full or nearly full motor recovery after anterior shoulder dislocation with brachial plexus injury of all included cases within 20 months is described, except for the intrinsic muscles of the hand. Intrinsic muscle recovery may be better in a younger age group (less than 50 years) [8]. They also conclude that nerve exploration is usually unnecessary. However, reconstructive surgery for the residual neurological deficit can provide improvement of hand function [8]. This might also explain the rather poor outcome in our patients compared to the current literature. On the one side, our collective is a lot older compared to those in the current literature, and on the other, our follow-up might miss the good outcome of the patients.

In contrast to the literature [13], electrographic examination (EMG or NCV) was carried out in only 23 (38.3%) of our patients. Although a quantification of nerve injury with the help of EMG or NCV is recommended in the literature, there is also the need of clinical examination of sensory and motor functions [3]. In addition, electrographic examinations are regarded as simple, inexpensive and widely applicable compared with other diagnostic methods, such as the wide range of imaging. Our low rate of EMG and/or NCV might be explained by the long study period and, therefore, the change of treatment algorithms over time. Nowadays, electromyography as well as ultrasound and MRI when indicated are standard investigations following shoulder dislocation with suspected lesions of the brachial plexus or its corresponding nerves in the treatment guidelines at the included centres (Fig. 1).

The final follow-up examination was performed after an average of 7 months, ranging from 1 to 86 months. This rate is insufficient which might be explained by the fact that over 70% of our patients presented with an age over 50 years. These patients may have lower requirements in daily life and, therefore, skip follow-up appointments. However, even in the literature, long-term follow-up studies are missing.

### Limitations

This study has several limitations. First, being a retrospective study, it is subject to multiple limitations inherent to this type of study design. In addition, data were derived from three different centres, which led to a further discrepancy in the retrospective comparison and the low incidence numbers might be skewed by inattentive documentation.

No correlation analyses were possible in this study; however, a good foundation for further studies in this field has been set.

## Conclusion

A combination injury of shoulder dislocation and brachial plexus lesion may occur at any age and sometimes carries a poor outcome. Electroneurographic examinations should be implemented in the management in future as a cost-effective and supportive examination.
